# Project MIMIC (Maximizing Implementation of Motivational Incentives in Clinics): A cluster-randomized type 3 hybrid effectiveness-implementation trial

**DOI:** 10.1186/s13722-021-00268-0

**Published:** 2021-10-12

**Authors:** Sara J. Becker, Cara M. Murphy, Bryan Hartzler, Carla J. Rash, Tim Janssen, Mat Roosa, Lynn M. Madden, Bryan R. Garner

**Affiliations:** 1 Center for Alcohol and Addiction Studies, Brown University School of Public Health, Box G-S121-5, Providence, RI 02912 USA; 2 Addictions, Drug, & Alcohol Institute, University of Washington, 1107 NE 45th Street, Suite 120, Seattle, WA 98105 USA; 3 Calhoun Cardiology Center - Behavioral Health, UConn Health, 263 Farmington Avenue, Farmington, CT 06030 USA; 4 Roosa Consulting, LLC, 3 Bradford Drive, Syracuse, NY 13224 USA; 5 APT Foundation, 1 Long Wharf Drive, Suite 321, New Haven, CT 06511 USA; 6 RTI International, 3040 E. Cornwallis Rd.Research Triangle Park, P.O. Box 12194, Durham, NC 27709 USA

**Keywords:** Contingency management, Opioid use, Addiction, Implementation, Addiction technology transfer center, Motivational incentives

## Abstract

**Background:**

Opioid-related overdoses and harms have been declared a public health emergency in the United States, highlighting an urgent need to implement evidence-based treatments. Contingency management (CM) is one of the most effective behavioral interventions when delivered in combination with medication for opioid use disorder, but its implementation in opioid treatment programs is woefully limited. Project MIMIC (Maximizing Implementation of Motivational Incentives in Clinics) was funded by the National Institute on Drug Abuse to identify effective strategies for helping opioid treatment programs improve CM implementation as an adjunct to medication. Specific aims will test the impact of two different strategies on implementation outcomes (primary aim) and patient outcomes (secondary aims), as well as test putative mediators of implementation effectiveness (exploratory aim).

**Methods:**

A 3-cohort, cluster-randomized, type 3 hybrid design is used with the opioid treatment programs as the unit of randomization. Thirty programs are randomized to one of two conditions. The control condition is the Addiction Technology Transfer Center (ATTC) Network implementation strategy, which consists of three core approaches: didactic training, performance feedback, and on-going consultation. The experimental condition is an enhanced ATTC strategy, with the same core ATTC elements plus two additional theory-driven elements. The two additional elements are Pay-for-Performance, which aims to increase implementing staff’s extrinsic motivations, and Implementation & Sustainment Facilitation, which targets staff’s intrinsic motivations. Data will be collected using a novel, CM Tracker tool to document CM session delivery, session audio recordings, provider surveys, and patient surveys. Implementation outcomes include CM Exposure (number of CM sessions delivered per patient), CM Skill (ratings of CM fidelity), and CM Sustainment (number of patients receiving CM after removal of support). Patient outcomes include self-reported opioid abstinence and opioid-related problems (both assessed at 3- and 6-months post-baseline).

**Discussion:**

There is urgent public health need to improve the implementation of CM as an adjunct to medication for opioid use disorder. Consistent with its hybrid type 3 design, Project MIMIC is advancing implementation science by comparing impacts of these two multifaceted strategies on both implementation and patient outcomes, and by examining the extent to which the impacts of those strategies can be explained by putative mediators.

*Trial registration*: This clinical trial has been registered with clinicaltrials.gov (NCT03931174).

Registered April 30, 2019. https://clinicaltrials.gov/ct2/show/NCT03931174?term=project+mimic&draw=2&rank=1

## Background

### Rationale for implementing contingency management in opioid treatment programs

The opioid overdose epidemic remains one of the most urgent public health crises in America’s history. According to the most recent National Household Survey of Drug Use and Health [1], about 1.6 million Americans 12 years of age or older met criteria for an opioid use disorder in 2019. Over 770,000 Americans have died from drug overdoses since 1999, and nearly 70% of all overdoses are due to opioids [2]. The societal costs of opioid use disorders extend well beyond mortality: opioid use disorders are linked to high rates of morbidity, disease transmission, increased health care consumption, crime and law enforcement costs, and lost productivity [3–5].

Medication for opioid use disorder with methadone or buprenorphine represents the front-line treatment for opioid use disorder [6–8], but medication alone is not sufficient for many patients. Even when treated with the newest extended-release formulations of medication [9], only about 40% of patients stay abstinent from opioids during the first 6-months of treatment [7, 10] and many patients struggle with treatment retention [11–13]. Recognizing the need to enhance medication’s effectiveness, the National Institute on Drug Abuse’s strategic plan [14] and the National Institutes of Health’s Helping to End Addiction Long-term initiative [15] have both emphasized the critical need for research to improve implementation of behavioral interventions as an adjunct to medication for opioid use disorder.

Contingency management (CM; i.e., providing patients with tangible motivational incentives for attaining pre-defined treatment goals) is one of the most effective adjunctive behavioral interventions in combination with medication for opioid use disorder [16–19], but one of the least available in opioid treatment programs (OTPs) that dispense medication and in other community settings. Surveys of front-line treatment providers suggest as few as 10% use CM [20]. These low rates of CM implementation reflect at least four distinct barriers. First, OTP providers are often unfamiliar with CM. In recent interviews with 43 OTP providers in Rhode Island, the investigative team found that only 42% were able to define CM correctly [21]. Second, OTP providers often object philosophically to the idea of incentivizing patients with tangible reinforcers for meeting treatment goals [22, 23]. An early survey found that over 50% of community providers objected to rewarding patients for attaining abstinence if they were failing to meet other treatment goals [22]. Third, organizations with a weak implementation climate (e.g., organizational stress, low openness to change) have worse attitudes related to CM implementation [24]. Fourth, and most critically, organization-level issues such as time and financial investment required to offer reinforcers are major barriers for OTPs seeking to implement and sustain CM [23, 25, 26]. In a study of 60 OTP providers followed for a year, the investigative team found that those providers who did not implement CM reported organizational-level barriers far more often than provider- or patient-level barriers [27].

By far, the most effective large-scale CM implementation effort to date was conducted through the Veteran’s Administration: years after implementation, over 90% of the initial agencies continue to deliver CM [28]. The key success factor in this initiative was institutional funding and commitment to ongoing training throughout an integrated system [28]. Efforts to implement CM in OTPs that lack the organization-level resources of the Veteran’s Administration system face a host of additional contextual barriers. For these reasons, multi-level strategies are likely needed to promote CM implementation in OTPs, to address both provider-level (e.g., knowledge, skills, attitudes) and organizational-level (e.g., implementation climate, time, funding) factors.

### Rationale for evaluating implementation strategies

Core to advancing implementation science is the recognition that evidence-based intervention delivery must be complemented by evidence-based implementation strategies [29]. Just as evidence-based interventions require specification of mechanisms of action and core components, so do implementation strategies. Yet, such specification remains rare in the field of implementation science. The current protocol aims to advance the field, by selecting two specific implementation strategies, enumerating them carefully, and conducting rigorous analysis of their comparative effectiveness.

The Addiction Technology Transfer Center (ATTC) network, funded continuously by the Substance Abuse and Mental Health Services Administration since 1993, represents the longest standing network of intermediary/purveyor organizations to help addiction treatment and recovery organizations to implement evidence-based interventions [30]. The New England ATTC—which serves the states of Connecticut, Maine, Massachusetts, New Hampshire, Rhode Island, and Vermont—uses a state-of-the-art training strategy consisting of didactic training, performance feedback, and on-going consultation [31, 32]. This approach targets provider-level factors (e.g., provider knowledge, skill, and attitudes). The combination of didactic training, performance feedback, and on-going consultation has been tested in multiple studies of staff training models and has been found to be more effective than self-study or individual components [33, 34]. As such, we selected the ATTC strategy as our “control” implementation strategy, since it is an empirically supported implementation strategy used in routine practice.

The investigative team has demonstrated that receipt of the ATTC strategy is associated with significantly greater odds of adopting CM than didactic training alone: in a study of 60 front-line providers in methadone clinics followed for 52 weeks, odds of adopting CM were 13.2 times greater in the ATTC condition than the didactic training only condition [35]. However, it took 4–5 months for the ATTC strategy to demonstrate superior effects and effects started to diminish after removal of active support [27, 35, 36]. For this reason, additional strategies may be needed to accelerate and sustain implementation of CM. Prior implementation research by our team supports the effectiveness and cost-effectiveness of both Pay-for-Performance [37, 38], and Implementation & Sustainment Facilitation [39, 40] to improve implementation and client outcomes relative to didactic training, feedback, and on-going consultation. The Pay-for-Performance strategy accelerates implementation via the extrinsic motivation of implementing staff members, whereas the Implementation & Sustainment Facilitation strategy promotes sustainment by drawing on the intrinsic motivation of the broader staff of a treatment organization. Given the urgent need to reduce the number of opioid-related overdoses and fatalities, we developed an enhanced implementation strategy that includes both the extrinsically motivating Pay-for-Performance strategy and the intrinsically motivating Implementation & Sustainment Facilitation strategy.

## Specific aims

Project MIMIC (Maximizing Implementation of Motivational Incentives in Clinics) is a cluster-randomized, type 3 hybrid trial focused on testing the effectiveness of the ATTC implementation strategy versus the enhanced-ATTC strategy. Both the ATTC and enhanced-ATTC strategies are guided by the Exploration, Preparation, Implementation, Sustainment (EPIS) model [41], a comprehensive implementation framework that can be used as both a process model to delineate the phases of implementation and a determinant model to highlight key contextual factors influencing implementation. Consistent with the EPIS framework, both implementation strategies delineate activities across phases (as elaborated in the “Implementation strategies” section). To elucidate factors driving implementation effectiveness, two inner context factors from EPIS that have been found to influence implementation effectiveness across multiple studies—implementation climate and leadership engagement [42, 43]—are tested as putative mediators [44].

Project MIMIC’s protocol is guided by three specific aims:

- **Specific Aim 1**, the primary aim, is to compare the effectiveness of the two implementation strategies on implementation outcomes. It is hypothesized that, relative to OTPs receiving the standard-ATTC strategy, those receiving the enhanced-ATTC strategy will demonstrate superior: (a) CM Exposure (i.e., patient-level measure of number of CM sessions), (b) CM Skill (i.e., staff-level measure of CM quality), and (c) CM Sustainment (i.e., patient-level measure of number of CM sessions received after removal of active support).
- **Specific Aim 2**, the secondary aim, is to compare the effectiveness of the two implementation strategies on patient outcomes. It is hypothesized that patients in programs receiving the enhanced-ATTC strategy will demonstrate: (a) more abstinence from opioids (i.e., days of abstinence), and (b) fewer opioid-related problems, relative to patients treated in programs randomized to the standard-ATTC strategy.
- **Specific Aim 3**, an exploratory aim, is to evaluate the extent to which two putative mediators reflecting inner context factors (i.e., implementation climate, leadership engagement) explain the impacts of strategy (i.e., ATTC vs. enhanced-ATTC) on implementation outcomes.

**Fig. 1:**
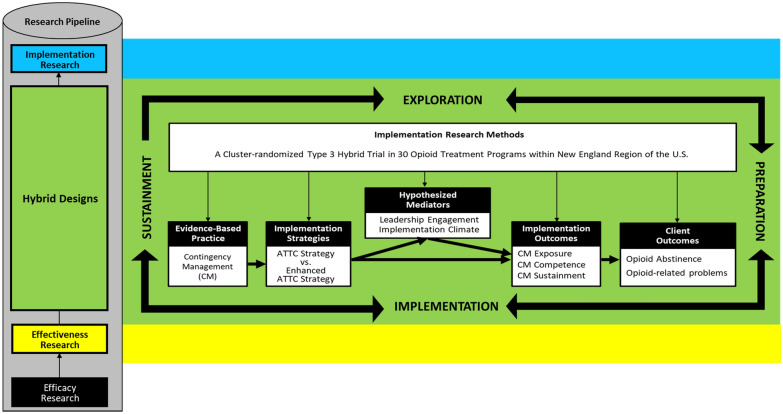
Conceptual overview of Project MIMIC (maximizing implementation of motivational incentives in clinics). Project MIMIC is a type 3 hybrid effectiveness-implementation trial guided by the Eploration, Preparation, Implementation, and Sustainment framework

Figure 1 presents the relationship a conceptual overview of Project MIMIC in line with the EPIS framework. This figure depicts the efficacy to implementation pipeline and highlights the different outcomes assessed in the study, as recommended in Proctor’s Conceptual Model of Implementation Research [45].

## Methods

### Study design

#### Hybrid trial

This protocol uses a type 3 hybrid effectiveness-implementation design [46], which prioritizes evaluation of implementation effectiveness (i.e., effect of the enhanced-ATTC and ATTC implementation strategies on implementation outcomes) as the Primary Aim and evaluation of clinical effectiveness (i.e., effect of CM on patient outcomes) as the Secondary Aim. Consistent with best practices for hybrid trials, a type 3 hybrid trial was selected because CM has robust evidence as an adjunct to medication for opioid use disorder [17, 18], yet it is underutilized and in need of effective implementation strategies to promote its utilization within OTPs. Considering the extensive data in support of CM [17, 18], an implementation trial focusing solely on implementation outcomes may have been justified [46]. However, a type 3 hybrid trial was selected to enhance the relevance of project findings to policy and practice [47], especially since implementation research has demonstrated that superior implementation outcomes do not always translate into better patient outcomes [48].

#### Cohort design

In order to maximize research efficiency and distribute research activities across the five-year study, the hybrid trial also utilizes a three-cohort staggered cluster randomized controlled trial design. A total of 30 OTPs will be enrolled across three cohorts, each consisting of 8–12 OTPs. Each cohort of 8–12 OTPs will receive 14 months of active support (consisting of preparation and implementation activities) and will then be followed for 6 months of ongoing monitoring (consisting of sustainment activities), as elaborated in the “Implementation strategies” section.

### Randomization

Following enrollment in the project, each OTP will complete a Baseline Organizational Assessment to gather site-level background data (e.g., years in operation, number of new patients served annually, number of staff, specific medications dispensed). Using these data, urn randomization procedures assign OTPs to the ATTC strategy (control condition) or the enhanced-ATTC strategy (experimental condition). For each cohort, the data core at the prime institution will create an urn randomization spreadsheet and research staff will create a spreadsheet listing organizational ID numbers with variables to be entered into the urn. One of the Multiple Principal Investigators, blind to the organizational ID numbers, enters each organization into the urn randomization spreadsheet to determine condition assignment. The urn randomization procedure balances on up to three variables per cohort: the most likely variables will include number of patients served, state in which the OTP is located (to account for disparate regulations across states), and percent of patients receiving different medications. Specific variables will depend on the characteristics of OTPs enrolled (e.g., if all the OTPs in a specific cohort dispense methadone then that variable would not be entered into the urn). OTPs, OTP staff, and CM trainers will not be blinded to study condition, but OTP patients and CM rating staff will be.

### Recruitment and informed consent

Reflecting the multi-level nature of the trial, participants will be recruited at three levels.

#### OTPs (organization-level)

A total of 30 OTPs in the United States will be enrolled in the project. The list of participating OTPs will be updated on an ongoing basis and maintained on clinicaltrials.gov (NCT03931174). Flyers and brief PowerPoint presentations about the project will be disseminated to Departments of Health in each of the six New England states, with a request to distribute to OTPs. Leaders of OTPs will be allowed to self-nominate by contacting research staff. To participate, OTPs must meet these criteria: (a) dispense either methadone or buprenorphine; (b) employ at least two counselors; and (c) enroll at least one new patient per week on average. OTPs that meet criteria will be enrolled until each cohort is filled (maximum of 12 OTPs). Upon enrollment, OTPs will be asked to sign a non-binding Organizational Agreement form, which documents the types of supports that the organization will receive and outlines the time commitment required.

#### OTP counselors and leaders (provider-level)

OTPs that choose to participate will nominate and provide the contact information of up to five front-line counselors and two leaders to participate in the study. Eligible counselors must have active caseloads of patients on medication and be willing to engage in CM implementation support. Leaders must supervise OTP counselors with active caseloads and be willing to oversee counselor engagement in CM training and implementation support.

Once nominated, counselors and leaders will be emailed an electronic consent form that describes the risks and benefits of participation, and outlines the study procedures. Research staff will conduct follow up phone calls with each of the nominated counselors and leaders to ensure they received the information and have an opportunity to ask any questions, prior to obtaining informed consent. An OTP must have a minimum of one leader and two counselors consent to participate in order to move forward. Across the 30 OTPs, the study aims to enroll up to 60 leaders (2 leaders per program × 30 programs) and 150 counselors (5 counselors per program × 30 programs), though actual numbers enrolled may be higher due to anticipated staff turnover at the OTPs. Staff turnover plans are discussed in the Staff Turnover Section.

#### OTP patients (patient-level)

Each OTP will be asked to refer at least 25 patients over a 6-month period for enrollment. Eligible patients will be adults (18 years of age or older) who were newly inducted on medication for opioid use disorder within the past 30 days and are currently receiving treatment at a partner OTP. The study focuses on newly inducted patients as opposed to those established in treatment because the need is highest among these patients: drop-out rates and missed medication doses are higher during the first 6 months of treatment (i.e., the “induction phase”) than any subsequent period [13]. Moreover, focusing on newly inducted patients was compatible with state and federal regulations for licensed OTPs at the start of this protocol [49] [prior to the novel coronavirus 2019 disease (COVID-19)]), which required new patients to receive more frequent (i.e., typically weekly) counseling early in treatment, thereby providing a natural opportunity for CM.

Newly inducted patients will be referred to the study by OTP staff during routine intake procedures. Patients interested in participating will complete a consent-to-contact form on a study-provided tablet at the OTP, which will give research staff permission to contact them about the study. Study procedures will be explained, including risks and benefits, and patients will have the opportunity to ask questions before signing an electronic consent form.

### Prize-based CM intervention

The prize-based CM intervention implemented in this protocol is the evidence-based, lower-cost prize model pioneered by Nancy Petry and colleagues, which has been tested throughout the National Institute on Drug Abuse’s Clinical Trial Network [28, 50, 51] and used in the Veteran’s Administration implementation effort [52]. The Petry model uses prizes of varying magnitude to reinforce patient behavior and has been shown to be effective when targeting abstinence, attendance, and other treatment goals [53]. Based on qualitative feedback with 11 OTPs using user-centered design principles [21], this study uses a customizable CM protocol targeting attendance, in which each OTP can develop an organization-specific definition of patients’ weekly attendance goals. The attendance target must include verifiable clinical encounters such as receipt of medication doses, completion of individual counseling sessions, and/or completion of group counseling sessions. The target may vary by stage of treatment but must be consistently applied to all patients within an OTP. For instance, one OTP might choose to define the weekly target as attendance at one group counseling session and one individual counseling session for the first month of treatment, and then only one group counseling session for the second month of treatment. The flexible, collaborative design approach used in this protocol has previously been shown to support sustainment of CM in the OTP setting [54].

Patients will earn prize draws weekly for meeting the attendance target. Draws start at one for the first demonstration of the attendance target and increase by one for each consecutive week that the behavioral target is attained. Failure to attain the benchmark resets draws to zero, with draws returning to one for the next on-target week and again escalating by one for every week in which the target is met. Patients will receive prize draws for up to 12 weeks. This progression yields a maximum of 78 prize draws (1 + 2 + 3 +…+ 12). Patients draw from fishbowls containing 500 slips of paper; 250 have encouraging phrases but are not associated with a prize, 209 state “small prize,” 40 state “large prize” and one states “jumbo prize.” Based on these probabilities and magnitudes of $1–2, $25, and $100 for the three respective prize levels, each draw has an average cost of $2.83. Thus, for a 12-week protocol, the average expected maximum for patients consistently meeting the attendance benchmark is about $220 in prizes (78 draws × $2.83/draw). Counselors will be given 14 weeks to complete the 12-week protocol to accommodate for excused absences and/or extenuating circumstances (12 session maximum). In response to the COVID-19 pandemic, all of the resources needed to conduct CM—fishbowl, prize menus, photos of the prizes—were provided to OTPs electronically so that CM could be delivered remotely as needed.

## Implementation strategies

**Fig. 2:**
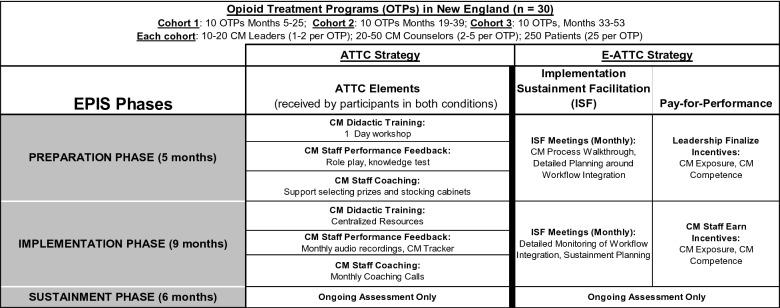
Overview of implementation strategies by phase of the Exploration, Preparation, Implementation, and Sustainment (EPIS) model. The exploration phase occurred in prior work with opioid treatment programs, as documented in Becker et al. 2020, BMC Health Affairs

Guided by the EPIS framework, which conceptualizes implementation as a “process,” [41] implementation activities in both conditions are divided into four phases. The Exploration phase was completed in recent formative work with OTP counselors and leaders, soliciting their preferences for CM implementation [21, 55]. Both strategies consist of both a 5-month Preparation phase and a 9-month Implementation phase. After an OTP has received these elements, it moves into a 6-month Sustainment phase. Detailed descriptions of each strategy are provided below by phase and presented visually in Fig. 2. OTPs in both conditions are asked not to enroll in any additional CM training during their receipt of the project’s active CM implementation supports.

### ATTC strategy

The ATTC strategy consists of didactic training, performance feedback, and on-going consultation. The ATTC strategy is only provided during the project’s preparation phase and implementation phase. All of the components of the ATTC strategy can be flexibly delivered in-person or virtually.

#### Preparation phase (5 months)

At the start of this phase, participating CM providers (counselors and leaders) are asked to complete surveys to assess their perceptions of CM, implementation climate, and implementation leadership at their OTPs.

##### Didactic training

In a training-to-criterion approach, OTPs will receive a didactic training workshop on CM. The typical workshop is a 7-h face-to-face training day. In response to the COVID-19 pandemic, the ATTC team successfully adapted the training into a series of three 1–2 h virtual training sessions (1 h of which is self-paced and 4 h of which are delivered synchronously), which will be offered as an alternative if social distancing orders prohibit in-person training. The didactic workshop is required for CM counselors and recommended for leaders. Continuing education credits are provided for completion of the training workshop.

The first half of the workshop consists of didactic instruction in CM principles, review of videotaped exemplar CM sessions, and small group discussion of how to develop a CM implementation plan. During the second half of the workshop, the trainer provides live demonstrations of CM delivery and providers break into pairs to complete role plays using standardized patient scenarios. Role-plays cover a range of possible scenarios including situations in which CM providers would have to describe CM to a new patient, provide reinforcement to a patient meeting the attendance target, and withhold reinforcement from a patient who did not meet the attendance target. Participants also practice scoring CM sessions for fidelity with the CM Competence Scale (described further in “Performance feedback” section). At the end of the training, attendees complete a 20-item CM Knowledge Scale modeled after the tool used in the Veteran’s Administration [28], which requires attendees to demonstrate an a priori CM knowledge criterion of 75% regarding application of CM principles to case vignettes. This criterion was established since scores of 70–80% are routinely required for continuing education credit [56, 57]. Counselors who do not pass the knowledge test are asked to take it again: those who do not receive a passing score two times in a row receive detailed corrective feedback.

##### Performance feedback

A similar training-to-criterion approach is applied to observed provider skills in CM delivery. A well-validated CM fidelity scale and coding manual developed by Petry and colleagues, the CM Competence Scale [58, 59], is provided during the workshop and used to guide provision of performance feedback on CM fidelity. As outlined in the scale, a CM session is expected to contain six key elements: (a) inform patient of reinforcement (i.e., number of draws) earned; (b) inform patient of reinforcement possible at the next session; (c) administer the appropriate number of fishbowl draws; (d) assess patient’s desire for prizes; (e) compliment or praise patient’s efforts toward attending treatment; and (f) tie attendance and the CM program to abstinence and other treatment goals. Providers are also expected to convey three general skills: (a) demonstration of general competence, expertise, and commitment; (b) maintenance of session structure; and (c) empathy. Items are scored on 7-point Likert scales: an average score of 4.0 out of 7.0 across the nine items is considered an adequate fidelity score [58], and serves as the a priori skills-based criterion demonstrating initial readiness to deliver CM.

Following the CM workshop, all OTP counselors who intend to provide CM receive performance feedback on one practice case. Counselors have 1 month following the workshop to submit an audio recording of a role-played CM session via encrypted email. Role-plays use a standardized patient scenario and are rated by blind coders for fidelity using the CM competence scale. CM counselors who demonstrate fidelity as indicated by an average score of “adequate” on the CM Competence Scale receive positive performance feedback. Those counselors who do not demonstrate satisfactory fidelity are given corrective feedback and OTP leaders are encouraged to provide additional training. OTP leaders receive copies of the summary reports distributed to counselors at their OTP to gain familiarity with using the CM Competence Scale as a supervision tool.

##### On-going consultation

In this phase, each OTP receives external consultation via email to help select a list of prizes, stock the prize cabinets, and finalize the organizational attendance target.

#### Implementation phase (9 months)

This phase is 9 months in duration and marks the start of CM implementation: OTPs recruit patients to enroll in CM for the first 6 months and have 3 months to finish the 12-week CM protocol. At the midpoint and end of this phase, providers (counselors and leaders) repeat measures of their perceptions of CM, implementation climate, and leadership engagement.

##### Training workshop

During the implementation phase, videos of the initial training and a number of CM resources (e.g., written case vignettes, standardized case descriptions, videotaped examples, orientation session example script, lists of materials required, etc.) are made available for review on a centralized ATTC website. Materials are available in English and Spanish. To allow for maximum flexibility (and to accommodate social distancing orders enacted during the COVID-19 pandemic), case examples and videotaped role-plays include CM delivered via telehealth, and resources are provided to enable remote session delivery (e.g., virtual fishbowl, electronic prize menus). Providers also receive a weekly electronic CM newsletter that contains tips for CM delivery and tracking, as well as a weekly CM trivia question.

##### Performance feedback

Throughout this phase, counselors delivering CM receive performance feedback via the CM Tracker [60], a web-based tool developed by the investigative team. More specifically, the CM Tracker helps prompt counselors to complete a brief Weekly Report for each CM patient. Each report takes 2–3 min to complete and collects: date of the CM session; target number of sessions patient needed to attend to earn prize draws; actual number of sessions attended; number of CM draws administered; and specific draws received (e.g., praise, small prize). It also contains the first 6 items of the CM Competence Scale [59], which counselors complete as a self-reported fidelity check. Data input into the CM Tracker yield a user-friendly dashboard for counselors that shows each patient’s progress through the CM intervention, summarizes fidelity of CM delivery, and automatically calculates the number of draws earned and number of draws anticipated in the next session. In parallel, leaders receive a user-friendly summary to quickly monitor their counselors’ delivery and fidelity of CM.

##### On-going consultation

The CM trainer and the New England ATTC Director (one of the project Multiple Principal Investigators) will co-lead monthly virtual CM consultation meetings. These meetings offer OTP providers (counselors and leaders) an opportunity to ask questions about how they can improve their CM technique(s). These meetings do not focus on higher-level implementation issues, such as implementation climate and leadership engagement.

#### Sustainment phase (6 months)

The focus of this phase is on having OTPs sustain CM without active support from Project MIMIC. OTPs continue to have access to all of the didactic CM-related resources via a centralized website. OTP leaders are encouraged to provide ongoing performance feedback to CM counselors as part of their ongoing operations, and CM counselors are encouraged to document CM delivery in their standard case records.

### Enhanced ATTC

OTPs randomized to the enhanced-ATTC experimental condition receive all aspects of the ATTC strategy described above, plus they receive both the Pay-for-Performance strategy and the Implementation & Sustainment Facilitation Strategy. As with the ATTC strategy, enhanced-ATTC strategy components can be delivered in-person or virtually.

#### Pay-for-performance

OTP counselors implementing CM earn monetary bonuses when they meet/exceed the performance goals for either of the project’s key performance measures: CM Exposure and CM Competence. These bonuses are only available during the implementation phase. Based on the Pay-for-Performance strategy shown to be effective and cost-effective as part of prior implementation research [36, 37], CM counselors earn US $200 for each newly admitted study-enrolled patient that receives 10 or more CM sessions (i.e., CM Exposure) within 14 weeks. Additionally, CM counselors earn $50 each month they attain an average score ≥ 5.8 on the CM Competence Scale, which corresponds with a “stretch” goal of strong CM skills. These benchmarks are empirically derived from prior CM randomized clinical trials with rigorous skills monitoring in which patients attended at least 80% of sessions [61] and highly trained counselors attained average competence ratings of 5.8 [62]. All payments are processed remotely and mailed via check within 30 days of attaining the benchmark.

#### Implementation & sustainment facilitation

Beginning in month three of the project’s preparation phase and continuing through the implementation phase, the OTP’s leaders and CM counselors participate in monthly 30–60 min Implementation & Sustainment Facilitation strategy meetings via Zoom. These meetings are facilitated by a trained facilitator who is external to the OTP. In accordance with its guiding theory, framework, and principles (see www.ISFstrategy.org for more information), each strategy meeting seeks to engage the OTP’s staff working on the project (i.e., the leaders and counselors who consented to participate), focus them on the project’s key preparation-phase outcomes (e.g., completing the didactic training, demonstrating CM proficiency), evoke from them the pros and cons of implementing CM, and plan how best to successfully transition from the preparation phase to the implementation phase. The Implementation & Sustainment Facilitation strategy’s decisional balance and past implementation effort exercises (see www.ISFstrategy.org/tools-resources/) are introduced and used as part of the preparation phase meetings, and are used during subsequent phases based on the OTPs needs. Near the end of the Preparation phase, each OTP is offered a longer 2 h CM process walkthrough meeting with the facilitator, which can be held either in person or virtually.

Each month of the implementation phase, the OTP’s leaders and CM counselors continue participating in 30–60-min virtual strategy meetings with their facilitator. Although introduced briefly during the preparation phase, it is during each of the implementation phase meetings that the Implementation & Sustainment Facilitation Strategy Workbook and embedded exercises (e.g., the performance review, evaluation, and planning exercise; implementation climate evaluation exercise; see www.ISFstrategy.org/tools-resources/ for demos) are used to help engaged, focus, evoke, and plan how best to maximize CM implementation both during the project’s implementation phase and beyond (i.e., the project’s sustainment phase). Given inadequate funding has been found to be a key barrier to CM implementation [25, 63, 64], a key part of planning efforts is focused on ensuring adequate financial support for CM. For example, the facilitator seeks to help leadership evaluate a range of options to financially support CM, including strategies to increase OTP revenue (e.g., fund-raising, seeking grant support, improving insurance collection rates, increasing patient fees, increasing patient flow), strategies to decrease OTP costs (e.g., improving operational efficiency, renegotiating contracts), and strategies to decrease the CM-related costs (e.g., seeking prize donations).

### Provider turnover plan

Consistent with implementation research highlighting the importance of training *and* retaining staff to competently deliver evidence-based practices [65], the effectiveness of both strategies is dependent on the ability to minimize and/or address staff turnover among the OTP’s staff working on the project. As such, our investigative team developed a staff turnover plan as part of the initial funding proposal that focuses on training as many counselors as possible, ensuring training materials are transferrable, and developing clear skills-based metrics for study participation.

Provider turnover research conducted by the investigative team [65–67] estimates an annual turnover rate of approximately 30% among substance use disorder front-line counselors and 20% among OTP leaders. OTPs are asked to nominate up to two leaders and five counselors to provide assessment data, but there is no upper limit on the number of leaders and counselors who can attend the didactic training, consultation calls, or strategy meetings. All didactic training and consultation activities are video- or audio-record so leaders have easily transferrable, low-cost training materials; this is consistent with usual practices at the New England ATTC, which routinely records training events. Recommended training of replacement counselors consists of watching/listening to recordings of didactic training sessions. Most critically, replacement CM counselors are required to attain the same a priori benchmarks on the CM knowledge test and CM Competence Scale as initial counselors, before they are approved to implement CM. Any time a role-play or knowledge test is submitted by a replacement counselor, leaders from that OTP are copied on the performance feedback to ensure they are familiar with the training and replacement process.

## Study outcomes

### Sources of data

**Fig. 3:**
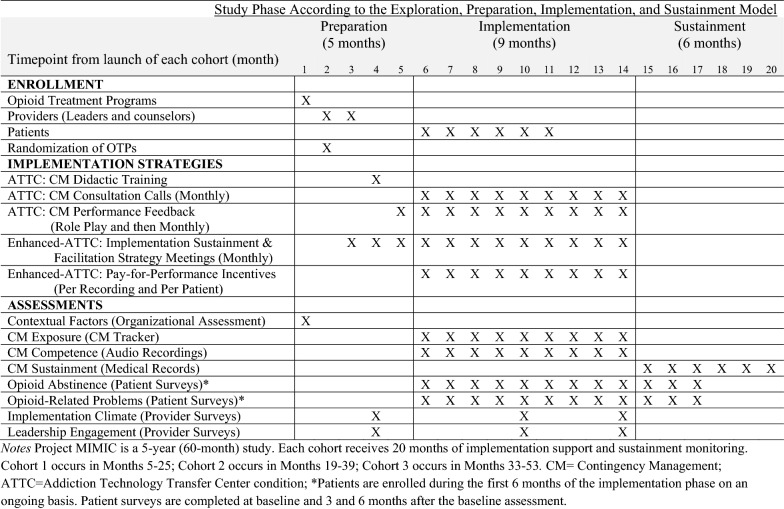
Study activities occurring in each cohort of Project MIMIC

Focal study measures include implementation outcomes (Aim 1), patient outcomes (Aim 2), and putative mediators (Aim 3). Data are collected via multiple methods, as elaborated below. The timing of data collection using these methods and the specific outcomes assessed are depicted in Fig. 3.

#### Organizational and provider surveys

Prior to randomization, the OTP’s designated leadership staff completes an organizational background survey to assess organization-level contextual determinants (e.g. leadership engagement, implementation climate). CM providers (i.e., counselors and leaders) complete three online surveys to assess contextual determinants at the provider level: at the start of Preparation, midpoint of Implementation, and end of Implementation. Providers receive $20–25 per completed survey.

#### CM tracker

Providers enter data regarding CM implementation into the CM Tracker on a weekly basis. Information entered into the Weekly Report provides data on the number of providers delivering CM per OTP, the number of sessions delivered per patient, and the number of prize draws administered. Data entered into the CM Tracker is cleaned by research staff weekly and research staff follow up with CM counselors if data entry lags are identified.

#### Audio recordings

On a monthly basis, CM counselors in both conditions are invited to submit an audio recording of a CM session for performance feedback. The audio recordings are rated with the CM Competence Scale and counselors in enhanced-ATTC have the opportunity to earn a $50 performance bonus.

#### Patient assessments

Patients complete brief surveys administered by research staff over the phone or via electronic link at baseline, 3, and 6-months post-baseline. Patients receive compensation for completed assessments in the form of a rechargeable Visa gift card (which is only activated after the patient confirms receipt) of $20–25 for each assessment.

### Retention of participants

To promote retention and encourage the completion of follow-ups, best practices in longitudinal trials are used. For instance, the trial employs a study identity (e.g., outreach efforts will have a consistent look with a study logo and study color pallet); a sense of community (e.g., providers will receive a weekly Project MIMIC newsletter with links to resources); systematic outreach (e.g., a set schedule to outreach to each research participant); escalating incentives (e.g., increase in remuneration over time); and comprehensive tracking methods (e.g., participants will complete detailed locator forms with multiple forms of contact).

### Primary aim: implementation outcomes

The primary aim tests the effectiveness of the two strategies (ATTC vs. enhanced-ATTC) on three implementation outcomes: CM Exposure, CM Competence, and CM Sustainment.

#### CM exposure

Measured at the patient-level, this outcome refers to the number of CM sessions delivered per patient during the 9-month Implementation phase. Data are extracted from the weekly records entered in the CM Tracker. For each patient, the number of CM sessions completed during their first 14 weeks of treatment is recorded (possible values ranging from 0 to 12), as well as whether they received the target level of CM exposure (10 + sessions). The target level of CM exposure is based on empirically derived benchmarks from prior effectiveness research [61]. The number of patients receiving CM and the number of counselors delivering CM will also be tabulated as indicators of exposure.

#### CM competence

Measured at the provider-level, this outcome is a measure of skill in CM implementation during the project’s 9-month Implementation phase. Each month, CM staff are asked to submit one CM session audio recording for rating by project coders (blinded to study condition) using the CM Competence Scale [59]. The CM Competence Scale contains nine items scored from 1 to 7, with higher scores indicating higher competence. A score will be created for each provider who submits a CM session recording on a monthly basis as well as whether the provider met the target level of CM competence (5.8), based on empirically derived benchmarks from RCTs [62].

#### CM sustainment

Measured at the organizational-level, CM sustainment will be measured using a variety of indicators including a count of the number of patients who received CM during the 10-month Sustainment phase, a count of the number of counselors delivering CM, and a count of the number of CM sessions delivered per patient. Leaders extract these data from their OTP’s medical records.

For both CM exposure and CM sustainment, we will adjust analyses to account for each program’s census to obtain a proxy of the intervention’s reach, which Glasgow et al. defined as the absolute number, proportion, and representativeness of individuals who are willing to participate in a given initiative, intervention, or program [68].

### Secondary aim: patient outcomes

The secondary aim tests the effectiveness of the two strategies (ATTC vs. enhanced-ATTC) on two patient outcomes: Opioid Abstinence and Opioid-Related Problems.

#### Opioid abstinence

atient self-report of abstinence from opioids will be assessed via 3- and 6-month post-baseline interviews, modeled after the well-validated Timeline Followback Interview [69]. Patients view a calendar and are asked: “*During the past 30 days, on how many days have you used opioids, painkillers, or other analgesics (excluding methadone and buprenorphine taken as prescribed)?*” and *“During the past 30 days, on how many days have you used heroin?*” to determine opioid abstinence. Corroboration of self-reported abstinence is attained via weekly reports of urine screen results entered in the CM Tracker. In each weekly report entered in the CM Tracker, providers indicate the date of any opioid urine screening, and whether the results were negative or positive for opioids.

#### Opioid-related problems

Opioid-related problems are assessed via patient self-report at 3- and 6-months post-baseline using the Opioid Problem Index, a count of 11 DSM-5 symptoms of an opioid use disorder experienced over the past month. This scale is based on the well-validated Substance Problem Index [70] from the Global Appraisal of Individual Needs [71], tailored to focus specifically on problems due to opioids.

### Exploratory aim: mediation

The exploratory aim focuses on the extent to which two organization-level variables (i.e., implementation climate, leadership engagement) mediate the relationship between implementation condition (i.e., ATTC vs. enhanced-ATTC) and each outcome in the primary and secondary aims. The putative mediators are collected from provider surveys administered to both counselors and leaders at the start of the Preparation phase and the midpoint of the Implementation phase.

#### Implementation climate

A 6-item measure of perceptions regarding the extent to which the innovation being implemented (i.e., CM) is expected, supported, and rewarded within the OTP. Each item is scored on a 5-point Likert scale. This well-validated, brief measure was developed by Jacobs and colleagues [72], and following published guidelines, we calculate a scale mean for each counselor and leader.

#### Leadership engagement

A 4-item measure of perceptions regarding whether leadership is committed to, involved in, engaged in, and accountable for implementation of the innovation (i.e., CM) at their OTP. Each item is scored on a 7-point Likert scale and a mean score is calculated for each counselor and leader. This measure has demonstrated excellent internal reliability (alpha = 0.94) and has been shown to be a significant predictor of staff-level implementation fidelity (β = 0.18, p < 0.05) [73].

## Data analysis, monitoring, and dissemination

### Data analysis

All analyses will be conducted following intent-to-treat principles [74, 75], such that all randomized study participants will be retained in analysis. All data will be reviewed for errors (e.g., range checks). Missing data will be handled following recommendations of the National Research Council [76]. Appropriateness of assumptions underlying use of missing data handling will be tested using sensitivity analyses [77].

For the primary and secondary aims, multilevel models will be tested in which patients (Level 1) are embedded within staff (Level 2) and within OTPs (Level 3). Consistent with Raudenbush and Bryk’s multilevel modeling approach [78] as implemented in a multilevel structural equation modeling framework [70], the proportion of variance to-be-explained at each level will be examined as an initial step. Next, following a decomposed-first strategy that advocates for starting with moderation-focused hypotheses to avoid biases associated with conflated effects [79], multilevel regressions will be conducted testing the extent to which our covariates significantly moderate the hypothesized relationship between condition and outcome. Covariates will be tested at three levels: organizational (e.g., years of operation, census/number of patients, medications prescribed, implementation readiness), counselor (e.g., tenure, education, CM attitudes), and patient (e.g., age, race, biological sex, problem severity). As noted previously, to obtain a proxy of reach, analyses of CM exposure and CM sustainment will be adjusted for OTP’s overall census of patients. If moderation is not found, covariates will be controlled for as predictors. Condition assignment (ATTC vs. enhanced-ATTC) will be the primary independent measure for analyses. In addition to reporting statistical significance, effect size estimates will be reported using Cohen’s *d* [80].

For the Exploratory Aim, a series of multilevel models will again be used. For each mediator, the average correlation within group (r_wg_) will be computed to determine whether aggregation of staff level responses to the organizational level is warranted. Values of r_wg_ range from − 1.00 to 1.00, with values ≥ 0.60 representing acceptable agreement [81]. Exploratory analyses will apply a mediation approach building on the work of Preacher and colleagues [82]. As part of this approach, the outcome will be regressed on the condition variable (ATTC vs. enhanced-ATTC) in a multi-level path model. In addition, the outcome will be predicted by both condition and the group mean centered mediator (e.g., implementation climate). Third, the mediational effect will be estimated as the confidence intervals of the bias-corrected bootstrapped product term of the effect of condition on the mediator and the effect of the mediator on the outcome variable at the between-group level. This method allows the between-group mediation effect to be partitioned from the within-group mediation effect.

### Sample size estimates

Power analyses were conducted to determine sample sizes and estimated power to test the effect of enhanced-ATTC, relative to ATTC, on the three implementation outcomes and two patient outcomes. All analyses assumed an alpha of 0.05 and an intra-cluster correlation coefficient of 0.05 at the organization level. Repeated measures analyses assume patient attrition of 20% and auto-correlations of 0.7. Analyses in Optimal Design Plus Software [83] indicated that there is sufficient power to detect small effect sizes on each outcome (*d*’s ≤ 0.37), according to Cohen’s taxonomy.

### Access to data

During active data collection, access to data is limited to the study investigative team. After the study has been completed, data will be available upon request from the Principal Investigators. Products created as part of this protocol such as a virtual prize bowl, training videos, and rating manuals, will be available publicly on a study website.

### Data protections

Efforts to protect the confidentiality of enrolled patients against the risk of unauthorized disclosure include the following: (a) all research staff are trained to understand the need for privacy, (b) all study information are stored in locked file cabinets located in access-controlled research offices, (c) study materials containing information about participant identity (e.g., electronic consent) are stored separate from the rest of participant data, (d) a unique research ID is assigned to each participant, (e) only the unique research ID is included as part of participant data, (f) participant names and contact information are stored in an electronic password protected master linkage file that is backed-up nightly, (g) data extracted from medical records is not linked to patient identifying data, (h) data are only reported in aggregate form, and (i) audio recordings are only listened to by research staff for the purpose of rating competence, and are erased at the end of the project.

### Data monitoring

This study is conducted under the auspices of the Brown University IRB, which conducts random audits of all IRB-approved protocols to ensure that the rights of human subjects are protected. The IRB reviews and approves any protocol amendments: approved amendments are reported to the sponsor in annual study updates and shared via clinicaltrials.gov on an ongoing basis. In addition, a Data and Safety Monitoring Board is used, which consists of experts in implementation science, contingency management, and multi-level modeling. The Board convenes annually independent of the sponsor and reviews protocol amendments and study adverse events.

Minimal risks are associated with the study and are limited to potential discomfort answering questions and breach of confidentiality. Adverse events are reported to the Multiple Principal Investigators within 24 h. Adverse events are reported to the institutional review board within 1 week of the Multiple Principal Investigator’s awareness of the event, with serious adverse events reported within 24 h. On an annual basis, the Board makes recommendations about trial continuation based on a detailed report summarizing trial progress, interim analyses of trial outcomes conducted by a statistician blind to treatment condition, and a running list of adverse events. An OTP’s participation in this protocol ceases after completion of the sustainment phase. No provisions for ancillary or post-trial support are planned, though OTPs can request technical assistance from the ATTC.

### Data dissemination

Irrespective of the magnitude or direction of effect, study findings will be widely disseminated. Planned dissemination efforts include presentations at professional scientific conferences and publication in peer-reviewed journals. In addition, results will be shared at the Addiction Technology Transfer Center’s annual leadership meeting to inform how the network of training and technical assistance centers delivers support to OTPs.

## Discussion

This protocol paper describes how two multifaceted strategies for CM implementation are being experimentally tested as part of Project MIMIC. Given the alarming rate of lethal opioid overdoses in the United States, which have continued to increase throughout the COVID-19 pandemic, strategies to advance the implementation and sustainment of effective adjunctive behavioral interventions to medication for opioid use disorder are urgently needed. This protocol is focused on comparing a credible real-world implementation strategy used by the largest intermediary/purveyor organization in the United States to an enhanced version of this strategy that addresses putative mechanisms of change (e.g., implementation climate, leadership engagement) that have been identified as critical components of the implementation process [84, 85].

Several design considerations guided this protocol each of which are associated with potential limitations. First, the enhanced-ATTC strategy contains two organization-level strategies that were specifically selected based on their focus on both the intrinsic and extrinsic motivation of implementing staff at the partner OTPs. While theoretically driven, if results favor enhanced-ATTC, it will not be feasible to empirically determine whether the results are driven by Pay-for-Performance or Implementation & Sustainment Facilitation. Assuming that the current protocol’s enhanced-ATTC strategy were found to be more effective than the ATTC strategy, subsequent research could seek to both replicate and expand upon the current project’s results via use of a 2 × 2 factorial design comparing: ATTC only, ATTC + Pay-for-Performance, ATTC + Implementation & Sustainment Facilitation, and ATTC + Pay-for-Performance + Implementation & Sustainment Facilitation. Important to note, careful consideration was given to this alternative design for use as part of the current protocol, but a 2 × 2 factorial design was not feasible without exceeding the funder’s limits on project costs.

Second, this protocol is focused on OTPs in New England and the demographics of patients receiving medication for opioid use disorder in New England, and of providers delivering it, tends to be less racially and ethnically diverse than in other parts of the United States. The investigative team decided to retain the focus on New England to align with the catchment area of the New England ATTC, yet committed to conducting aggressive outreach and recruitment efforts to recruit as diverse and representative of a sample as possible. Finally, Project MIMIC has a 6-month sustainment phase, which limits conclusions about the longer-term impacts of these strategies.

The limitations of Project MIMIC are outweighed by several strengths including: (a) the rigorous design as a sequential cohort cluster randomized trial, (b) the collection of outcomes from multiple sources and levels including organizational records, data entered into a novel tracking tool, session audio recordings, and patient assessments, (c) focus on the high-need setting of OTPs, (d) large number of organizations with 30 OTPs each enrolling up to seven counselors and leaders, (e) examination of multiple phases of the implementation continuum (preparation, implementation, and sustainment), (f) testing putative mediators, and (g) partnership with a regional training and technical assistance center that can translate the results into action.

Results of this protocol have the potential to inform how the entire network of regional ATTCs funded by the Substance Abuse and Mental Health Services Administration, as well as other relevant intermediary purveyor organizations, help OTPs to implement effective interventions. Such work could potentially enhance retention in OTPs and thereby enhance the quality of care offered to patients receiving medication for opioid use disorder who are at high risk of lethal overdose and other serious harms. Additionally, findings will help advance the field of implementation science by experimentally comparing different strategies and by exploring the extent to which the impacts of these strategies are able to be explained by implementation climate and/or leadership engagement.

## Data Availability

The datasets that will be generated from this protocol will be available from the corresponding author on reasonable request.

## Abbreviations

ATTC: Addiction technology transfer center
CM: Contingency management
EPIS: Exploration, preparation, implementation, and sustainment
OTP: Opioid treatment program
MIMIC: Maximizing implementation of motivational incentives in clinics
